# US and EU radiopharmaceutical diagnostic and therapeutic nonclinical study requirements for clinical trials authorizations and marketing authorizations

**DOI:** 10.1186/s41181-019-0059-2

**Published:** 2019-05-22

**Authors:** Sally W. Schwarz, Clemens Decristoforo

**Affiliations:** 1 Washington University School of Medicine, St. Louis, MO USA; 2 Department of Nuclear Medicine, Medical University Innsbruck, Innsbruck, Austria

**Keywords:** Radiopharmaceuticals, Regulation, FDA, EMA, Preclinical safety, Toxicity testing

## Abstract

New regulatory guidance documents from the United States Food and Drug Administration (FDA) and the European Medicines Agency (EMA) have recently been finalized or are in draft format outlining new pathways for preclinical safety testing. The US and the European Union appear to be moving in a similar direction focussing and refining preclinical safety data requirements for both radiodiagnostics and radiotherapeutics. We here summarize these recent documents from both the US and European perspective.

## Introduction

New regulatory guidance documents from the United States (US) Food and Drug Administration (FDA) and the European Medicines Agency (EMA) have recently been finalized or are in draft format outlining new pathways for preclinical safety testing. The US and the European Union appear to be moving in a similar direction focussing and refining preclinical safety data requirements for both radiodiagnostics and radiotherapeutics. We here summarize these recent documents from both the US and European perspective.

### What are the new non-clinical guidance documents recently developed in the US?

The US FDA Diagnostic Guidance, Microdose Radiopharmaceutical (RP) Diagnostic Drugs: Nonclinical Study Recommendations (Microdose Radiopharmaceutical Diagnostic Drugs, n.d), intends to refine nonclinical study recommendations for diagnostic RP drugs given its unique characteristics which include microdosing and single or infrequent clinical use. It reduces or eliminates additional toxicology requirements, and clarifies other non-clinical requirements for microdose diagnostic RPs for Phase 1–3 studies. Additionally the guidance helps sponsors facilitate timely conduct of clinical trials, reducing the use of animals, use of drug development resources, and provides recommendations for a pathway to full drug development [marketing authorization (MA)] for microdose RP diagnostic drugs. This guidance will hopefully reduce the requirements without comprising patient safety. In the past, toxicology studies were performed in laboratories complying with Good Laboratory Practice (GLP). Recent changes in the US, through pre-IND meetings with the FDA, have allowed toxicology studies to be performed in other types of controlled laboratories such as university comparative anatomy or veterinary medicine departments which could further reduce non-clinical costs.

The US FDA has also published a separate Draft Guidance, Oncology Therapeutic Radiopharmaceuticals: Nonclinical Studies and Labeling Recommendations, for radiotherapeutics (Oncology Therapeutic Radiopharmaceuticals, n.d). It discusses evaluation of toxicities from systemic administration of the ligand, evaluation of radiation toxicities, and product labeling. The Guidance is intended to supplement the existing guidance which addresses Nonclinical Evaluation of Late Radiation Toxicity of Therapeutic RPs (Guidance for Industry, n.d). This guidance will assist sponsors in designing appropriate nonclinical studies before initiation of first-in-human (FIH) trials and then continue through product approval. It also provides recommendations for labeling, including discussion on the duration of contraception to minimize potential risk to a developing embryo/fetus and recommendation for lactating women to minimize potential risk to a nursing infant. The guidance provides recommendations for nonclinical programs in a unique and challenging area of product development, provides a more consistent approach for nonclinical studies and product labeling, and reduce the conduct of nonclinical studies that are not informative for product use.

### What are the comments on these new guidance documents from the EU?

European authorities have not released such dedicated guidelines intended to give guidance for marketing authorization of diagnostic and therapeutic radiopharmaceuticals (RPs). The US FDA Diagnostic Guidance, Microdose Radiopharmaceutical Diagnostic Drugs: Nonclinical Study Recommendations is aimed at the industry and therefore especially of interest for companies aiming at MA, where this document can be used especially to define later stages of development in discussion with the authorities, EMA and national drug agencies. For early phase trials especially in the academic setting, the document may be of limited use in Europe, for this dedicated guidance exists (see below). Also, non-GLP toxicology studies are so far not accepted in Europe and there is no indication at this moment that it will be. The recent FDA guidance on therapeutic RPs, even though intended as “guidance for industry” may stimulate similar activities in Europe. In contrast to the US, so far regulatory guidance in Europe has not made such a clear distinction between therapeutic and diagnostic radiopharmaceuticals. A difference to the US is that the academic clinical research is still a major driver in the development of new therapeutic RPs. These guidance for therapeutic radiopharmaceuticals could serve as a good reference also in Europe, however only in close discussion with the competent authorities. In the case of therapeutic radiopharmaceuticals, this will be EMA, rather than national regulatory agencies.

### What new developments are ongoing in the EU with respect to non-clinical safety testing?

For the translation of radiopharmaceuticals in Europe so far ICH(M3) guideline (ICH guideline M3(R2) on non-clinical safety studies for the conduct of human clinical trials and marketing authorization for pharmaceuticals, n.d) has to be followed and has been the main guidance available. Its limitations for radiopharmaceutical development were addressed in a position paper by the European Association of Nuclear Medicine (EANM) (Koziorowski et al., 2017). Recently EMA has published a draft Guideline, dedicated to RPs, on the non-clinical requirements for radiopharmaceuticals (Guideline on the non-clinical requirements for radiopharmaceuticals, n.d), on November 22, 2018. This guideline covers both radiodiagnostics and radiotherapeutics, and provides guidance for a targeted approach to assess the pharmacology and safety of the non-radioactive part of a radiopharmaceutical. It addresses the non-clinical evaluation, and in particular the toxicity testing requirements, as prerequisite for a clinical trial authorization as well as (finally) for an MA. In contrast to US guidance it does not address radiation induced toxicity, as this is covered by Directives of EURATOM (Directive 2013/59/Euratom). It stresses a risk based, targeted approach for a non-clinical programme for testing RPs to support clinical trials. Different scenarios were taken into account covering the high variability of radiopharmaceutical targeting structures used today. Example include minimal change in a known radiopharmaceutical structure, or how to deal with higher molecular weight species exceeding the 100 μg limit. Based on this guideline careful design of preclinical studies should allow simplified, less cost intensive translation of radiopharmaceuticals into the clinic. However, whereas pharmacological studies, including imaging and biodistribution studies, can be done outside GLP, for toxicity studies GLP is generally expected, unless a scientific justification is given, which should “..address the potential impact of the non (GLP)-compliance on the reliability of the safety data” and “..the study (is) run according to the principles of GLP as close as possible” (Guideline on the non-clinical requirements for radiopharmaceuticals, n.d). It should be stated that this guideline is still in draft status and may change once potential comments have been addressed.

### What are the US comments in regard to the EU guideline on the non-clinical requirements for RPs?

This new EU document is a very detailed guideline for both diagnostic and therapeutic studies, and they will assist the nuclear medicine community in the design of preclinical studies. As was mentioned above, the separate discussion for the ligand and for the radionuclide is well done, and will provide a reference for the US in discussions with the FDA for planning pre-clincial studies. The US Guideline is focused more toward industry and by contrast the EU Guideline is organized in a manner that will assist development of both diagnostic and therapeutic radiopharmaceuticals, in the academic arena as well as for industry.

In regard to the GLP practices regarding toxicology studies as discussed in the new EU non clinical requirements, it does state that “This guideline must be read in conjunction with … Directive 2004/10/EC on harmonization of laws … .to the application of the principles of good laboratory practice and the verification of their application for tests on chemical substances.” Compared to the US FDA recent thinking allows toxicology studies to be performed in other controlled laboratories which could further reduce non-clinical costs. It does seem that this approach should be considered in the new EU Guideline. Most university laboratories are fully aware of what is required for GLP or could work with a non-GLP toxicology laboratory to provide additional dose verification or other needed information to allow these studies to be performed less expensively.

### What about submission of human trial data, collected outside of the US or the EU, for use in submission of a marketing authorization?

Drug development has become a global initiative and it is important that data from multiregional clinical trials can be accepted by regulatory authorities across regions and countries as evidence to support marketing approval of drugs (medicinal products). The recent US FDA approvals of F-18 Axumin in 2016 and Ga-68 Netspot® in 2017, were supported by data from clinical trials in other countries. This was an important change from historic requirements of country specific clinical trial data collection.

In the US a new guidance “E17 General Principles for Planning and Design of Multiregional Clinical Trials (MRCTs) Guidance for Industry” was finalized in July 2018 (E17 General Principles for Planning and Design of Multiregional Clinical Trials, n.d). The purpose of this guidance is intended for planning and design of MRCTs with the goal of increasing the acceptability of data from global regulatory submissions. This potentially could reduce the cost and accelerate of drug development, and could assist in expediting translation of new diagnostic and therapeutic radiopharmaceutical development.

## Conclusion

Regulatory authorities both in the US and Europe seem to have recognized the need for a more specific approach for RPs in the translation from preclinical development to clinical application. A number of documents were recently released or are in a draft format. Hopefully these activities will lead to a more harmonized approach to simplify preclinical safety data requirements for a clinical trial submission, and potential MA for new RPs. These issues are recognized by nuclear medicine scientific societies such as the Society of Nuclear Medicine and Molecular Imaging or EANM e. These societies need to continue to advocate to the US FDA and EU EMA to assist in interactions between these countries to allow a more universal approach to the requirements for clinical trial conduct and MA submission which would assist in greater patient access to novel diagnostic and radiotherapeutic RPs.

## Abbreviations

EANM: European Association of Nuclear Medicine
EMA: European Medicines Agency
EU: European Union
FDA: (United States) Food and Drug Administration
FIH: First-in-human
GLP: Good Laboratory Practice
ICH: International Council for Harmonisation of Technical Requirements for Pharmaceuticals for Human Use
IND: Investigational New Drug
MA: Marketing Authorization
MRCT: Multiregional Clinical Trial
RP: Radiopharmaceutical
US: United States of America
